# AI, aging well, and accessible digital games: the supplemental role of AI in accessible game design for older adults

**DOI:** 10.1093/geront/gnag055

**Published:** 2026-04-26

**Authors:** Brandon Lyman, Yichi Zhang, Celia Pearce, Miso Kim, Casper Harteveld, Leanne Chukoskie, Bob De Schutter

**Affiliations:** College of Arts, Media and Design, Northeastern University, Boston, Massachusetts, United States; College of Arts, Media and Design, Northeastern University, Boston, Massachusetts, United States; College of Arts, Media and Design, Northeastern University, Boston, Massachusetts, United States; College of Arts, Media and Design, Northeastern University, Boston, Massachusetts, United States; College of Arts, Media and Design, Northeastern University, Boston, Massachusetts, United States; College of Arts, Media and Design, Northeastern University, Boston, Massachusetts, United States; Bouvé College of Health Sciences, Northeastern University, Boston, Massachusetts, United States; College of Arts, Media and Design, Northeastern University, Boston, Massachusetts, United States; Khoury College of Computer Science, Northeastern University, Boston, Massachusetts, United States

**Keywords:** Aged heterogeneity, Video games, Personalization, Threat-to-self-esteem, Age-friendly design

## Abstract

As the population continues to age, and gaming continues to grow as a hobby for older people, heterogeneity among older adult gamers is increasing. We argue that traditional *game-based* accessibility features, like simplified input schemes, redundant information channels, and increased legibility of digital user interfaces, are limited in the face of this heterogeneity. This is because such features affect all older adult players and, therefore, are designed generically. We introduce artificial intelligence—although it has its own limitations and ethical concerns—as a method of creating *player-based* accessibility features, given the adaptive nature of the technology. These features may help to address a unique assemblage of accessibility needs that may accumulate through age. We argue that existing AI technologies can build upon extant accessibility design techniques to improve digital game accessibility for heterogeneous older adults. We adopt insights from gerontology, human–computer interaction, and disability studies into the digital game design discourse for older adults, and we contribute insight that guides the integration of player-based accessibility features to supplement game-based counterparts. The accessibility of digital games for heterogeneous older adults is paramount, as the medium offers short-term social, emotional, psychological, cognitive, and physical benefits that support the long-term goal of aging well.

## Supporting aging well through accessible digital games: The supplemental role of AI in game design for older adults

More adults aged 50+ are playing games now than ever, and the average age of gamers is increasing (Entertainment Software Association [ESA], 2024). Older adults are an especially heterogeneous group in terms of health, ability, personality, and social participation (Dannefer, 1988; Grigsby, 1996; Light et al., 1996). It follows that growth in this population brings with it divergent accessibility needs that are difficult to address due to their bespoke nature.

Current approaches to digital game accessibility for older adults alone are not robust enough to handle this increased heterogeneity. Although they provide a baseline level of accessibility that is paramount for many older gamers to play games at all, they fall short in that they cannot possibly account for the more nuanced and individual-specific barriers to gameplay. As a result, the growing population of older adults may not be able to surpass such barriers and may miss out on many of the benefits associated with digital gaming, many of which support an individual’s ability to age well. We argue that a robust accessibility approach would encompass both game-based, general accessibility enhancements coupled with enhancements that are tailored to the unique needs of an older adult individual playing the game.

Artificial Intelligence (AI) is an emerging technology that has shown promise in improving accessibility outside of digital games (Chemnad & Othman, 2024; Hatami & Chegini, 2024; Mitre & Zeneli, 2024; Sain, 2024). Recent research suggests that AI can personalize accessibility experiences (Huang et al., 2024; Li & Liao, 2025; Mitre & Zeneli, 2024; Sain, 2024), which positions AI as a potential supplement to traditional accessibility solutions in digital games. Although this approach has its own flaws and brings with it ethical concerns, it could help to mitigate the accessibility issues at the level of the individual rather than the game.

**Figure 1:**
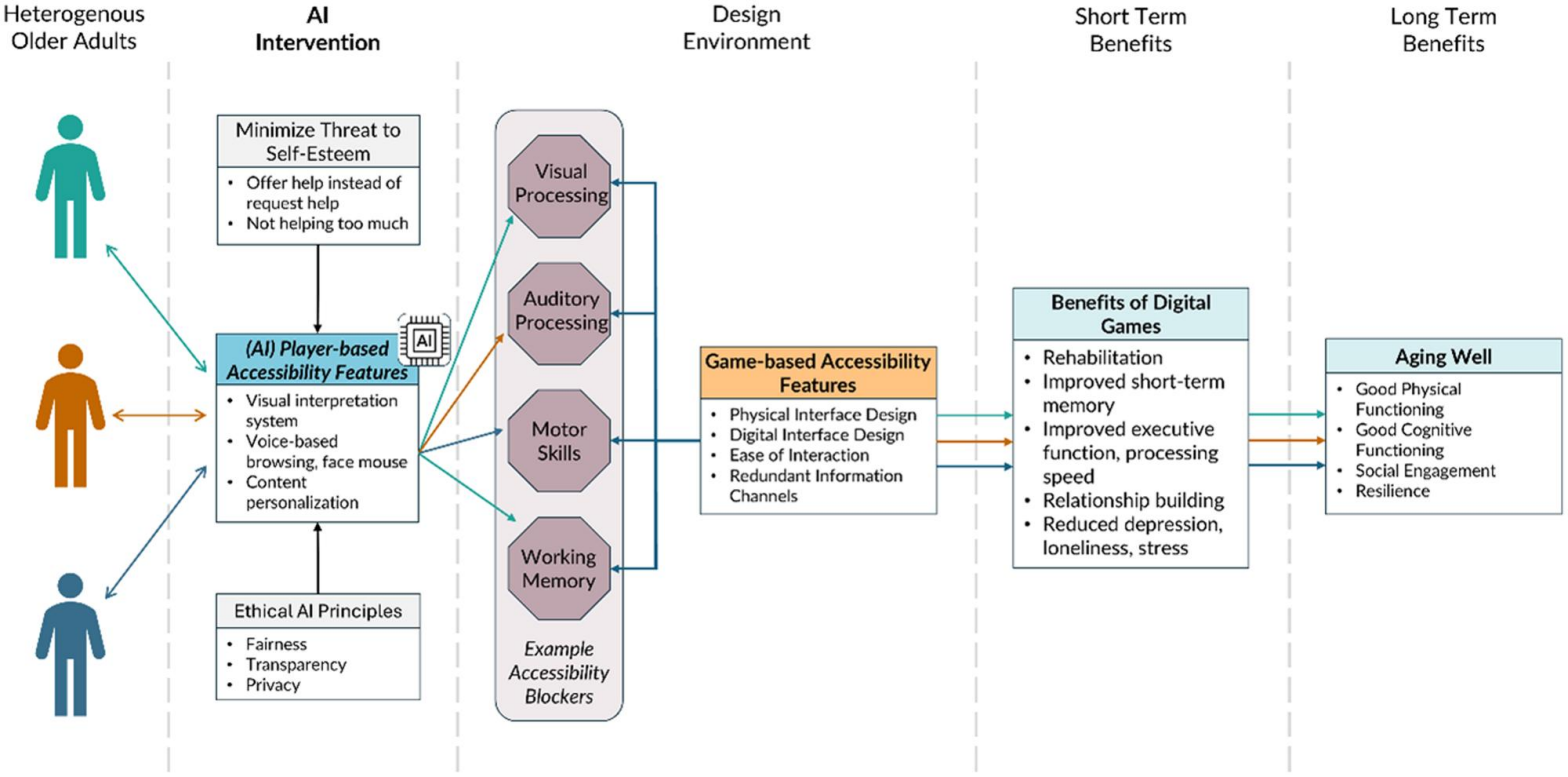
Conceptual player-based accessibility and a game-based accessibility map. A conceptual map showing the relationships between older adults, accessibility blockers, AI-driven, player-based accessibility features, game-based accessibility features, and the benefits of digital games. (1) Heterogenous older adults with unique accessibility needs (represented by different colors) interact with the player-based accessibility features. (2) Personalization capabilities of AI allow player-based features to operate within the unique context of the older adult and work to minimize threat to self-esteem (Fisher et al., 1982), guided by ethical AI principles. (3) The player-based features and the game-based features work to reduce accessibility blockers from the player side and design side, respectively. (4) Lowering these barriers allows older adults to obtain the short-term benefits of digital games (represented again by colored arrows), supporting the longer-term goal of aging well.

This article synthesizes the implications of growing heterogeneity among older adult gamers; the existing game-based approaches to accessibility, noting their merits and limitations; the benefits older adults can obtain from digital gameplay; and how those benefits are supportive of aging well. We contribute generative insight through examples from human–computer interaction that suggest a player-based, AI-driven approach to game accessibility may be achievable with existing applications. Finally, we discuss the broader implications of introducing AI to digital games and accessibility discourse for older adult gamers. An overview of our synthesis is provided in Figure 1.

## Heterogeneity in aging

Analysis of recent demographic trends shows that the global population is aging and will continue for the foreseeable future. A 2024 report noted that the number of people aged 60 and over will roughly double by 2050, from 1 billion in 2020 to 2.1 billion in 2050 (World Health Organization [WHO], 2024). The same report mentions that the proportion of the world population aged 60 and over will nearly double, from 12% in 2015 to 22% in 2050 (WHO, 2024), so the demographic is likewise growing compared to other demographics.

Alongside these global aging trends, there is evidence that older people are playing more digital games than ever before. The ESA (2024) reported that nearly 30% of all digital game players in the United States are over the age of 50, up from 17% in 2004 and 9% in 1999. Atop the frequency charts of why older adults choose to play games, 83% of Boomers and members of the Silent Generation who play games indicated they do so to pass the time or relax, 59% indicated they do so to have fun, and 61% indicated they do so to keep their brain and mind sharp (ESA, 2024). AARP (2024) reports that 45% of adults aged 50 and over play games at least once a month based on a 2023 tech trends report produced by the organization. The report also notes that many Gen Xers are already over 50, and millennials are not far behind; thus, they expect the growing trend of aging gamers to prevail (AARP, 2024).

Serving this growing population is challenging due to inherent diversity amongst older adults. The concept of aged heterogeneity is described in the late 1980s, in a paper that criticized the discourse at the time for ignoring a “seemingly obvious” phenomenon that people become more individualized as they age (Dannefer, 1988, p. 373). Building upon this foundation, Grigsby (1996) presents a short review of three studies that they feel properly capture aged heterogeneity in the domains of political economy, health, and personality. Finally, Light et al. (1996) investigate the drivers of aged heterogeneity from the life course perspective, concluding that heterogeneity is derived from the interaction between personality and social environments, concluding that people become “more who they are” as they age (p. 171).

Such heterogeneity influences a game developer’s ability to cater to the needs of this demographic. Blocker et al. (2014) note that personality traits such as introversion and extraversion may predict an older adult’s receptiveness to violent digital game content and contend that such personality traits may need to be considered to promote adherence to game-based interventions. Differences between the Western, individualist mentality and the more socially oriented East Asian cultures pose an interesting challenge as well; following insight from Hadi Mogavi et al.’s study, designing an exergame that is based on self-improvement is likely to appeal to older adults in Western cultural societies and unlikely to be appealing to those whose cultural roots are in East Asia (Hadi Mogavi, 2024). Even indicators such as age, gender, and education can influence how older adults perceive and interact with games, evidenced by Pinazo-Hernandis et al.’s (2025) finding that women age 60–70 with higher education and digital experience from their occupation were among the most frequent gamers in the study (2025). From these few studies, we see that demographic differentiators such as personality, culture, and past experience pose design problems for game developers. Game accessibility is an extension of the game design, and thus, this heterogeneity poses design problems for accessibility design.

Recognition of the growing older adult gamer population, coupled with the complexity introduced by aged heterogeneity, motivates our consideration of player-based accessibility solutions through AI to supplement existing game-based accessibility solutions described in prevailing digital games literature. We will explore these game-based solutions, their merits, and their limitations in the next section.

## Game-based accessibility features

We use the term *game-based* to describe accessibility features that are implemented into the digital implementation of the game itself. Accessibility-driven decisions that are implemented into the game influence all who play it, despite their age or unique performance abilities. The game design literature for older adults is primarily focused on improving the efficacy of these features.

For instance, Whitlock et al. (2011) suggest designing the game to be controlled by single-handed game controllers, which are more friendly toward age-related changes in physical ability. Additionally, they recommend implementing an adaptive difficulty scheme to help compensate for differences in player performance capabilities (Whitlock et al., 2011). Gerling et al. (2012) present an extended model of digital game design that encourages designers to consider age when designing their user interface and core mechanics. Vasconcelos and Silva (2012) propose ten “rules of thumb” for digital gaming platform design for older adults, which include ways of easing interaction, transportability to aid those with physical decline, and age-friendly interfaces (p. 8). Marston (2013) suggests that game interactions should be simple, controlled by voice or gestures, and “relate to real world or life experiences” when designing games for older adults (p. 114). Gamberini et al. (2014) provide a plethora of design implications for aging players of digital games, including utilizing redundant channels for transmission of crucial information, decreasing steps required to complete an action, and provision formal training or manuals to learn about the game (p. 298–301).

These accessibility design insights are critical in providing a baseline level of accessibility to many people with varying levels of physical and cognitive ability. Many encourage minimizing unnecessary complexity, which in our opinion is crucial to effective game design regardless. Such game-based features are a sound approach given the limitations of designing an entertainment product, especially for a more homogenous demographic such as teens and young adults. As heterogeneity increases with age, however, older adults’ accessibility needs to trend away from general and may require a more individualized approach to fully access digital games.

One way to conceptualize the importance of the accessibility features is through McLaughlin et al.’s (2012) notion of a cost-benefit analysis performed by older adults when deciding to play a game. In this model, the costs associated with playing digital games include lack of control, initial frustration, usability challenges, poor design for aging, and feelings of clumsiness, among others (p. 16). Many of these costs are directly associated with games’ accessibility and are reduced by the efforts of game designers to implement game-based accessibility features. Moreover, the model contends that older adults exhibit less motivation to play digital games if the costs outweigh the benefits. By considering additional, *player-based* accessibility features, we seek to further reduce the weight on the negative side of the scale such that benefits outweigh the costs.

## The benefits of digital gaming and aging well

On the other side of McLaughlin et al.’s (2012) cost-benefit scale are the benefits of digital gaming. Their article identifies self-esteem, physical activity, social interaction, and positive emotions as benefits that are considered by older adults in this equation (McLaughlin et al., 2012). Many of these benefits are consistent with the themes and goals of *aging well*, a concept coined by Waddell et al. (2024) that evolved from a synthesis of research on the concept of *successful aging*.

*Successful aging* is a term first coined by Rowe and Kahn (1997) to describe maintaining desirable physical, cognitive, and social functioning throughout the aging process. The original model prioritizes avoidance of disease and disability, high cognitive and physical function, and engagement with life as indicators of successful aging (Rowe & Kahn, 1997, p. 434). Several newer models of successful aging are built upon Rowe and Kahn’s foundational model, synthesized by Waddell et al. (2024), the result of which extends successful aging to include elements such as resilience, spirituality, and autonomy, among others. We refer the reader to Waddell et al.’s (2024) work for a more comprehensive understanding of their synthesis.

Waddell et al. (2024) highlight the flaws in the successful aging model, noting that the labeling of factors that are beyond the control of an individual (such as disease and disabilities) as success or failure is an unjust dichotomy (p. 6). Moreover, they contend that such a conceptualization of aging fails to consider individual context, which resonates with our proposal of *player-based* accessibility features supplementing game-based counterparts (Waddell et al., 2024).

To highlight the connection between digital gameplay and aging well, we briefly expand upon the benefits identified by McLaughlin et al., offering insight from more recent literature. Research suggests that digital games can provide beneficial effects for players, including social benefits, such as relationship building (Kaufman, 2017; Mohsin et al., 2021; Reer & Quandt, 2020); emotional benefits such as alleviation of depression (Kaufman, 2017), reduction of loneliness or isolation (Kaufman, 2017), leisure (Boot et al., 2020; Mohsin et al., 2021), and eliciting appreciation (Reer & Quandt, 2020); psychological benefits such as needs satisfaction, stress reduction, and personal fulfillment (Reer & Quandt, 2020); cognitive benefits such as subjective short-term memory improvements (Mohsin et al., 2021), executive function, and processing speed improvements (Zhang & Kaufman, 2016); and physical benefits, such as rehabilitation (Mohsin et al., 2021; Zhang & Kaufman, 2016).

These benefits are supportive of the elements of aging well discussed earlier. Creating new relationships via digital game communities helps older adults to continue engagement in social relationships, and this social support could likewise foster resilience to adversity. The emotional and psychological benefits are likewise supportive of the resilience required in the aging well framework, as they work to mitigate the impact of negative emotional responses while creating positive responses such as enjoyment or appreciation. Cognitive and physical benefits of digital games support good cognitive and physical function. From this synthesis, we contend that access to digital games supports an older adult’s ability to age well and that accessibility barriers to digital games prevent older adults from obtaining such benefits. More robust accessibility practices could reduce the costs of playing a game.

## The case for player-based accessibility features

Whereas *game-based* accessibility features describe those implemented in the game itself, *player-based* accessibility features instead improve accessibility in a personalized and adaptive manner. As they exist in games today, player-based accessibility features take the form of manual adjustments the player must make themselves and automatic adjustments that are made based on player actions or performance. A renowned example of manual adjustments is present in *Celeste*, a precision platformer with a deep, emotional story in which the player must summit a seemingly insurmountable peak. *Celeste* offers a granular accessibility suite known as “assist mode” that lets the player fine-tune their gaming experience to fit their abilities (Madson, 2023). Options include tweaks to the speed of the game, adjusting resource abundance, and even making the player invincible (Madson, 2023). Somewhere in between manual and automatic is *Halo 2* (2004), in which the tutorial asks the player to look up at a light in the game world. The player can either push their joystick up or down, which implicitly records whether they prefer default or inverted vertical camera controls. The player has in effect decided how they want the game to be controlled without opening a menu, though they can still use a menu to change that decision should they wish to do so. Finally, a common example of purely automatic player-based accessibility features is dynamic difficulty adjustment. In such an approach, the skills of the individual player are communicated to the system, and the system responds by changing the content to better suit those skills (Zohaib, 2018). While dynamic difficulty adjustment is not necessarily driven by AI, it is an example of the kinds of player-based systems we contend will benefit gamers as they age. Literature on AI-based accessibility specifically for older adults in digital games is scarce. Through review of tangential literature from human–computer interaction and AI studies, we see opportunities for digital game design discourse to adopt AI for creating more player-based accessibility features. Before discussing these opportunities, however, we wish to draw attention to the risks, ethical considerations, and limitations of implementing AI in this space.

### Risks & ethical considerations

In terms of risks to the individual, there are three pertinent negative consequences that could be experienced directly by users of AI accessibility solutions: lack of transparency, the potential for bias and discrimination, and data privacy risks.

The risks involved with lack of transparency are the inability to build trust between AI experts and users (Siau & Wang, 2020). Furthermore, any incomplete understanding of an AI algorithm leaves room for biases, as the natural complexity of the algorithm helps to obscure biases from view (Eitel-Porter, 2021, p. 74).

Bias and discrimination in AI algorithms are a result of the data they are trained on (Siau & Wang, 2020), which could include reflections of current and historical social inequalities (Shukla, 2024, p. 29–30). The data the algorithm is trained on could also contain gender bias or racial bias that informs the AI application (Siau & Wang, 2020). An example of bias being perpetuated in AI systems and pertinent to the demographic we are serving is ageism, which takes on several forms in the AI space, including the inclusion of bias in digital datasets and the exclusion of older users from AI technology (Stypinska, 2023). Hong and Choi evaluated responses from Open AI’s GPT-4o model to prompts about personality descriptions of ages 10–90 (2025). They found that older adults were depicted by the large language model as being more homogenous, less competent, and less positively assertive than younger populations, representing common, harmful stereotypes (Hong and Choi, 2026). Propagating these biases on a large scale could further harm marginalized populations (Shukla, 2024, p. 29).

Data privacy is a risk in the use of AI because such algorithms need copious amounts of training data to be effective, which can often be comprised of sensitive personal data (Zhou et al., 2020). As with any activity that involves collecting personal data, AI applications introduce a risk of privacy breach in which unauthorized users of the data leverage the information for unregulated and nefarious means, like identity theft (Piñeiro-Martín et al., 2023). More specifically to AI, the ability of the algorithm to recognize patterns could lead to the deduction of information it does not directly have access to, like sexual orientation (Stahl, 2021).

The discourse around ethical AI is vast, and a comprehensive understanding of the discussion is beyond the scope of this article. In short summary, ethical AI is achieved for the individual when (1) the system is *transparent* and *explainable* (Shukla, 2024); (2) its usage *does not discriminate* against users based on “race, religion, gender, sexual orientation, disability, ethnic (sic) origin, or any other personal condition” (Barredo Arrieta et al., 2020, p. 36); and (3) keeps the vast amount of user data needed to operate the model *private* (Wei and Liu, 2025).

### Limitations of AI in digital games

Beyond ethical drawbacks, there are several practical considerations when introducing artificial intelligence to digital games. First, unique to digital games is the derivation of fun as a result of overcoming tasks that are designed to challenge the user (Boot et al., 2020). We are discussing the use of AI to ease accessibility issues for older adult players. But games are designed to be difficult, and the introduction of AI assistance may compromise the intended difficulty of the game. While this may prove to be a difficult balance, it is important that an effective AI accessibility system does not over-help the player overcome game challenges.

Next, computational costs must be considered due to the constraints of modern technology. As alluded to in the previous section, AI algorithms require vast amounts of data to run, and computational costs of running AI algorithms require high-performance processing hardware (Talib et al., 2021). Since over half of all 50+ gamers prefer the mobile platform to more computationally capable devices (Kakulla, 2023), implementation of proposed AI solutions on platforms commonly used by this demographic may be hindered by smartphone processing speeds.

Finally, it is worth considering that commercial digital games are generally proprietary. Therefore, developers are protective of the game’s codebase. It is possible that an AI system would need to act directly on the game’s code to adapt it to the player, which is unlikely to be accepted by many digital game companies. AI-based adaptability may need to be endogenous to the game’s development process, limiting widespread adoption and driving up total implementation costs when adopted.

The drawbacks associated with the use of AI will need to be deeply considered before implementing any player-based accessibility features aimed at reducing accessibility concerns on a large scale. Meanwhile, it is generative to examine how existing AI solutions could be applied to accessibility in digital games. We will do so by describing specific accessibility issues and demonstrating how AI has helped address each issue in fields other than digital game design. While we acknowledge that many types of accessibility barriers exist, for scoping purposes we chose to focus on cognitive and physical accessibility barriers.

### Example 1: Visual and audio processing

Decline in sensory functions like vision and hearing can negatively impact a gaming experience. Impaired vision could make it more difficult to read text, identify objects, and detect game prompts. This could impair performance, engagement, and overall experience with digital games (Gamberini et al., 2014; Gerling et al., 2012; Rienzo & Cubillos, 2020; Vasconcelos & Silva, 2012). Reduced contrast sensitivity, slower visual adaptation, and slower processing of sensory information make it challenging for older adults to distinguish details in complex graphics or fast-moving scenes, as well as to adapt to changes in light intensities or contrasts in games (Harada et al., 2013). Additionally, reduced visual depth perception can make it hard for older adults to accurately estimate distances, specifically affecting their ability to interact with three-dimensional elements in games (Harada et al., 2013).

Compounding the challenges associated with the changes in visual function, hearing difficulties may present challenges to older adults when following verbal instructions, recognize audio cues, or distinguish between background music and important game sound effects. This can hinder the ability to accurately respond to audio-based tasks, leading to missed opportunities or slow reactions during gameplay (Cota et al., 2015). Taken together, the lower fidelity of visual and auditory information poses additional strain on the cognitive functions discussed earlier, as older adults may need to work harder to interpret visual or auditory information to compensate for sensory limitations, increasing cognitive load (Speekenbrink & Shanks, 2013), and potentially increasing the risk of cognitive decline (Maharani et al., 2018).

To address these accessibility concerns, a common use of AI in areas other than digital games is to make accessible visual information through audio. In a systematic literature review, Chemnad and Othman (2024) note that the majority of AI and accessibility discourse is focused on assisting with visual impairments. Kumar et al. (2024) provide a collection of on-the-market technologies to assist people with visual disabilities. They include often-used applications like virtual assistants (Google Assistant, Cortana, and Siri) but also more specialized technologies such as screen readers (TalkBack and VoiceOver) and a visual identification assistant called Lookout (Kumar et al., 2024). Abdolrahmani et al. (2018) investigated user perception of these technologies, concluding from inductive thematic analysis of semistructured interview responses that the assistive applications “enable participants to perform independently without the need for sighted assistance or specialized hardware/software” (p. 251). Another empirical study employing reflexive thematic analysis of semistructured interview and observation data found that a combination of Siri and VoiceOver had a strong, positive influence on maintaining independence for those with visual impairments (Sayago & Ribera, 2020). These findings suggest that, since existing technologies appear to be effective at assisting visually impaired people with nonspecific, daily tasks, similar technologies could be likewise effective in assisting those experiencing age-related decline in vision in a more specific setting such as digital gameplay.

Less prevalent are visual-based accessibility solutions for those with reduced hearing ability. Hatami and Chegini (2024) developed an AI-driven system that converts speech to images and emojis to assist hearing-impaired individuals with understanding spoken language. The ethical constraints imposed by the creators on model parameters, as well as the focus on providing responses as close to real-time as possible (Hatami & Chegini, 2024), bodes well for the future of adapting such systems to operate ethically in the often fast-paced digital worlds of modern games.

### Example 2: Motor skills

The decline in motor skills can have a wide impact on the operational aspects of gaming for older adults. Often this change presents an initial barrier to gameplay through the controller itself. For example, if the controller is very small, the buttons are not well spaced, or if the game requires buttons to be pressed simultaneously, the control may act as a barrier for older adult gamers (Gamberini et al., 2014). A decline in fine motor skills could limit an older gamer’s ability to make precise movements such as aiming or performing combos (Dick & Overton, 2010; Gerling et al., 2012).

Additionally, a decline in hand-eye coordination associated with age makes it more difficult for older adult gamers to perform actions quickly and accurately in games, thereby limiting overall performance (Cota et al., 2015; Gerling et al., 2012). Such a barrier could present itself in areas including operational precision, simultaneous actions (e.g., pressing multiple buttons or using dual joysticks), mistakes, and missed actions (Gamberini et al., 2014).

The specialized physical demands of modern digital games may prohibitively challenge the declining motor skills of some older adults. Madaan and Gupta (2021) describe the development of assistive technology that combines voice commands, gestures, and a face-controlled computer mouse. This technology uses natural language processing to allow the user to interact with web sites with their voice. Additionally, a bespoke convolutional neural network is used for detecting hand gestures, with up to 94% accuracy with 27 trained hand gestures (Madaan & Gupta, 2021). No AI or machine learning was required to create the face-controlled computer mouse (Madaan & Gupta, 2021). Technologies such as Madaan and Gupta’s could offer an alternative method of interacting with digital games rather than traditional input devices that require small and precise inputs, such as a keyboard and mouse.

### Example 3: Working memory

Working memory is defined as the “temporary storage and process information required for cognitive tasks such as comprehension, learning, and reasoning” (Engle & Kane, 2003). According to Baddeley’s (2000) model, working memory consists of four components correspondingly functioning to control related attention, retain verbal information, hold visual and spatial details, and integrate information across systems and time. This model suggests that declined working memory can present challenges in games, such as difficulty focusing on and switching tasks, forgetting instructions or dialogue, getting lost in visual environments, struggling to follow the storyline or combine clues, etc.

Games often require players to retain and process information to engage in play, and sometimes players need to interact with many pieces of information simultaneously. A decrease in working memory increases multitasking demand, which could induce feelings of frustration for older adult gamers (Cota et al., 2015; Gamberini et al., 2014; Gerling et al., 2012). Even when the player is not multitasking, working memory decline may also hinder an older adult gamer’s adaptability, especially when games require frequent task switching or decision-making. This could lead to a decrease in game motivation and enjoyment (Gerling et al., 2012; Rienzo & Cubillos, 2020). Additionally, reduced ability to analyze complex information may require older adults to expend more effort in processing and retaining information for multistep tasks or complex narratives, leading to more difficulty in decision-making processes (Dick & Overton, 2010).

Digital games also can demand intense working memory loads for some older adults. In an early development that could support working memory abilities in the future, Makhataeva et al. (2023) describe the implementation and user testing of an AI system (coupled with augmented reality) to help participants remember physical locations of objects. The study involved participants taking a walking tour of a 3-story lab facility. They were tasked with memorizing the location of several objects in the 3D space. The AI system was used to identify objects and map them to a 2D representation of the 3D space. Cognitive demand and effort greatly decreased with the use of the system, whereas temporal demand and frustration also decreased with a lower effect size (Makhataeva et al., 2023).

These findings suggest that the ability of AI systems to scan and interpret 3D visual information, as well as simplify these representations, can reduce the amount of cognitive effort required to complete spatial tasks. Additionally, this system was created with the Unity game engine, a publicly available tool for game development. This indicates that the adoption of this technology into a digital game environment could be straightforward, as it was developed using the same tools as digital games.

### Personalization

Key to the idea that AI could be used to create player-based accessibility features is the personalization capabilities exhibited by the technology. A survey in education found that AI technologies promote learning accessibility by creating personalized education paths for users with varied needs (Sain, 2024). Mitre and Zeneli (2024) drew similar insights from a literature review conducted on the topic of AI in higher education. Research on AI models such as ChatGPT has found that such models are capable of developing user personas autonomously for the purpose of adaptive user experience and user interfaces (Huang et al., 2024). This highlights the ability of AI to personalize a user experience based on data from the user. In a more direct application of AI, Li and Liao (2025) created a digital game about fishing that used AI to tailor the content of the game in accordance with the performance of the older adult player. The adaptive application was shown to improve satisfaction and willingness to play (Li & Liao, 2025). It follows from these studies that AI could theoretically tailor an accessibility approach to a player. The approach could address accessibility issues to the extent that the individual needs them addressed without the need to assume or diagnose any existing cognitive or physical issues. The system could accomplish this based on personas created from the player’s anonymous data in a way that addresses their unique accessibility needs.

## Aid and threat-to-self-esteem

As a final piece to the puzzle of player-based digital game accessibility features, we consider the threat-to-self-esteem model (Fisher et al., 1982). This consideration further complicates the goal of reducing accessibility barriers to digital games. The main considerations that apply here are (1) the characteristics of the entity providing the aid, the aid itself, and the perception of the aid as threatening or helpful; (2) if the aid is perceived as threatening, older adults may have a negative reaction such as lowered self-concept; and (3) a distinction between accepting an offer of aid versus requesting aid is important in predicting the impact on self-esteem (Fisher et al., 1982). In criticizing the threat-to-self-esteem model, Newsom (1999) proposes a relationship between five variables (helping characteristics, relationship variables, caregiver variables, care recipient variables, and situational variables), interactions with the caregiver, and negative reactions to care. A comprehensive understanding of each of these variables is outside the scope of this article; however, the key takeaway is that the inherent complexity of human relationships makes it difficult to predict whether a reaction to aid from another human will be positive or negative. This underscores the need for a personalized approach to offering help. Moreover, in an AI system an algorithm, rather than another person, would be offering this help. This is likely to reduce the influence of the caregiver, relationship, and situational variables described by Newsom.

AI seems to address this concern gracefully due to an AI model’s ability to act independently of manual input. Based on Huang et al. (2024) and Li and Liao’s (2025) experimental results, AI-driven changes to the game’s user experience could be executed without an explicit request from the user. The threat-to-self-esteem model predicts that this would lead to more positive reactions to assistance, as “aid that must be requested may elicit threat because it involves a public admission of inferiority, whereas an offer of aid does not” (Fisher et al., 1982, p. 40). Framing the activities of an AI accessibility agent as offering assistance would, therefore, be less likely to negatively affect self-image. This is preferable to the alternative of navigating a menu to search for the accessibility features needed, which could be viewed as a request for aid—or worse, requiring the assistance of another person to adjust the accessibility features for them, which would almost certainly be viewed as a request for aid.

More recent studies in human–computer interaction introduce more nuance to this discussion, however. In a study on intelligent voice assistants (IVAs) in the context of health information retrieval, Karimi and Martin-Hammond found that older adults saw potential in voice-controlled systems to make them lazier, which in the long term could make them less independent in reviewing their health information (2025, p. 19). The participants also noted that such laziness worked against their desire to remain healthy and to be active in maintaining their own health (Karimi and Martin-Hammond, 2025, p. 20). Further concerns arose in this study regarding the increase of dependence on the IVA being detrimental if the IVA ceases to be dependable (Karimi and Martin-Hammond, 2025, p. 20). Another study on health misinformation and older adults found that participants were interested in an intelligent chatbot acting as a mentor or educator on misinformation, helping them to improve their ability to think critically rather than simply protecting them from misinformation (Peng et al., 2024). This study further underscores the goals older adults have when using emerging technology: maintaining or improving independence. Another potential concern, as highlighted by Kim et al. in the context of digital games, is the level of anthropomorphism present in the intelligent assistance offered to the player (2016). The study found that anthropomorphizing the intelligent assistance systems led to lower enjoyment of the game and lower perceived autonomy when playing, as opposed to comparable intelligent systems that were not anthropomorphized (Kim et al., 2016). The author argues that lowered autonomy can threaten self-esteem following Fisher et al.’s model (Kim et al., 2016, p. 284). As these studies demonstrate, merely differentiating between asking and offering help is not sufficient in considering the design of these systems. Instead, the design of these systems needs to promote active engagement in the help process, act more as a mentor teaching independence than acting as a tool to be relied upon, and strive to avoid anthropomorphizing the intelligent assistance.

## Conclusion

From this analysis, we conclude that AI-driven applications have the potential to address accessibility concerns of older adults at an individual level, recognizing and respecting the heterogeneity of older adults and limiting negative reactions to aid predicted by the threat-to-self-esteem model. This would comprise what we consider to be a player-based approach to digital games accessibility for older adults, which we maintain should be implemented in addition to traditional game-based accessibility approaches prevalent in the literature. Although AI is not a brand-new technology, its entry into the modern zeitgeist is recent. The ethical and cultural impacts have not been studied to a great enough extent for us to recommend it as a solution to accessibility problems. More research is required to understand what designing a fair, private, and transparent AI solution for this space would look like. At a practical level, however, it seems to us that AI uniquely fits the demands of player-based accessibility solutions.

While we have focused on compensation for deficits associated with aging, we also note that the heterogeneity discussed in the older adult population is not only in terms of these deficits. Older adults may also possess relative strengths compared to their contemporaries or other age demographics. As such, it is important to note that future work could focus on how AI would adapt gameplay to further challenge those strengths just as much as compensate for weaknesses. This could improve an older adult’s ability to age well, with more challenging games improving resilience, autonomy, and cognitive function.

Further research is required on modern older adults’ perceptions of AI to understand whether such a technology would be successfully appropriated if developed. Additionally, research on safe and ethical AI implementation is required, as an AI-powered accessibility, as we have discussed, introduces several risks to users. Finally, older adults’ opinions on what an AI-powered accessibility solution for digital games would look like should be paramount, and we suggest that research on the design for such an application be grounded in participatory design methodologies.

## Data Availability

This article does not report data, and therefore, the pre-registration and data availability requirements are not applicable.
